# Editorial: Advances in Understanding of the Functions of the Paraventricular Thalamic Nucleus

**DOI:** 10.3389/fnint.2021.744147

**Published:** 2021-08-17

**Authors:** Seema Bhatnagar, Gilbert J. Kirouac

**Affiliations:** ^1^Department of Anesthesiology and Critical Care, Children's Hospital of Philadelphia Research Institute, The Perelman School of Medicine at the University of Pennsylvania, Philadelphia, PA, United States; ^2^Rady Faculty of Health Sciences, University of Manitoba, Winnipeg, MB, Canada

**Keywords:** thalamus, emotions, stress, reward, circuits

Formative work by Groenewegen et al. established in the early 1990's that the paraventricular nucleus of the thalamus (PVT) was a major source of afferent inputs to the nucleus accumbens of the ventral striatum (Berendse and Groenewegen, 1990, 1991). It was notable that PVT afferents overlapped within subregions of the nucleus accumbens with inputs from the prefrontal cortex, subiculum, and basolateral nucleus of the amygdala (Wright and Groenewegen, 1995, 1996). Discoveries of new peptides in the 1980's and early 1990's led to characterization of several peptides and receptors in the PVT (Kirouac, 2015). Despite the clear anatomical evidence demonstrating that the PVT is well-positioned within the ventral striatal circuits that regulate motivated behavior, the number of experimental studies on the PVT were infrequent compared to studies on the other inputs to the nucleus accumbens. The subsequent demonstration that the PVT also provides equally dense projections to the bed nucleus of the stria terminalis and the central nucleus of the amygdala further implicated the PVT in the regulation of emotional behaviors (Li and Kirouac, 2008).

It was evident in numerous studies using expression of the immediate early gene c-Fos that the PVT is a region of the brain strongly activated by emotionally arousing and stressful conditions [see Hsu et al. (2014)]. The first functional studies of the PVT emerged in the late 1990's demonstrating its role in regulation of stress responses (Bhatnagar and Dallman, 1998; Bhatnagar et al., 2000), energy metabolism (Purvis and Duncan, 1997; Bhatnagar and Dallman, 1999), and drug responses (Young and Deutch, 1998). In the past 20 years, interest on the PVT has expanded greatly with a number of studies firmly establishing that this area of the thalamus contributes to both emotional and motivated behaviors. For instance, the PVT has been shown to not only contribute to natural reward seeking and food intake responses as well as drug-seeking responses, it has also been shown to regulate aversive behavioral responses associated with the emotions fear and anxiety. Other lines of investigation demonstrate that the PVT is a critical component of the brain circuits involved in producing adaptive responses to stress.

The topic includes seven articles exploring the functional role of PVT including two new empirical contributions that examine the function of the PVT in a rodent model of drug-seeking and brain imaging of human subjects. The topic also includes five timely review papers that discuss some of the most significant experimental findings to date implicating the PVT in wide variety of appetitive and aversive behavioral response. We anticipate that this collection will provide those who are interested in studying the PVT a useful starting point for their own investigations.

Matzeu and Martin-Fardon further establish the contribution of the neuropeptide orexin in the PVT in reward-seeking in rats made dependent on ethanol by chronic intermittent vapor exposure. They show that pharmacological blocking of orexin receptors in the PVT prevented stress-induced reinstatement of ethanol and a food reward seeking in ethanol-dependent rats. These experimental findings are consistent with the view that the PVT integrates and relays stress-related signals to regions of the brain involved in modulating motivation and that exposure to addictive substances leads to maladaptive behaviors.

Kark et al. examine functional connectivity patterns of the PVT in resting human subjects using MRI imaging. These findings indicate that the PVT in human subjects is functionally coupled with many regions of the brain known to be structurally connected in rodents and non-human primates. The study suggests that the PVT is integrated into the default mode network associated with self-referential thought. This study provides foundational information for investigating how the PVT functions in human subjects.

Kooiker et al. discuss the potential contribution of the PVT by which early-life emotional experiences influence a wide range of behaviors throughout the lifespan and contributes to resilience or vulnerability to mental health disorders. The paper highlights what is known about the anatomy and function of the PVT in the regulation of behavior and discusses how novel technical approaches can be used to better understand how early-life experiences produce enduring behavioral changes by modifying PVT circuits.

Curtis et al. combine findings in the published literature with their observations from the Allen Brain Atlas to describe the distribution of neuropeptides in PVT neurons of the rat and mouse with a special focus on neuropeptides known to be involved in regulation of behavior. They observe that different neuropeptides are not evenly expressed across the anteroposterior axis of the PVT. They further discuss potential functional differences between the anterior and posterior aspect of the PVT based on the distribution of neurons expressing these neuropeptides in the PVT.

Kirouac describes the anatomical connections of the PVT with forebrain regions known to regulate aversive behaviors and discusses the evidence that shows that neurons in the PVT integrate viscerolimbic information and threat signals from diverse regions of the brain. Research findings are discussed demonstrating that stress can produce a long-lasting enhancement of orexin signaling in the PVT in a way that can lead to anxiety-like behavioral tendencies. These experimental observations along with recent anatomical discoveries are used to generate a model of how the PVT regulates anxiety-like behaviors.

Iglesias and Flagel review the significant findings implicating the PVT in the regulation of motivation including some of the differences between the anterior and posterior aspects of the PVT. There is a focus on the importance of a hypothalamic-thalamic-striatal circuit where it is posited that the PVT integrates information regarding the internal states and the external environment to translate it into motivated actions. They discuss their research findings that orexinergic innervation from the hypothalamus encodes the incentive motivational value of reward cues.

McGinty and Otis discuss the contribution of the PVT to behavior and highlight some of the contradictory findings reported in the various investigations. They discuss some of the afferent inputs and efferent projections by which the PVT regulates conditioned appetitive and aversive responses and the novel approaches that have been employed to study the PVT as well as some of their limitations. The authors discuss the fact that the PVT is anatomically complex and provide suggestions on how to further our understanding of the PVT's role in motivation.

The experimental findings and theoretical discussions regarding the function of the PVT clearly point to the need to further explore how the PVT fits within the brain circuits that mediate adaptive behavior including learned and motivated responses and those associated with stress adaptation. It is also apparent that the PVT is both neurochemically and anatomically complex and that the development of novel experimental approaches will be instrumental in furthering our understanding of the functions of the PVT.

## Author Contributions

Both authors were involved in writing this Editorial and approved it for publication.

## Conflict of Interest

The authors declare that the research was conducted in the absence of any commercial or financial relationships that could be construed as a potential conflict of interest.

## Publisher's Note

All claims expressed in this article are solely those of the authors and do not necessarily represent those of their affiliated organizations, or those of the publisher, the editors and the reviewers. Any product that may be evaluated in this article, or claim that may be made by its manufacturer, is not guaranteed or endorsed by the publisher.
